# Implementing a General Practice-Based Link Worker Intervention for People with Multimorbidity During the Covid-19 Pandemic- a Mixed Methods Process Evaluation of the LinkMM RCT

**DOI:** 10.5334/ijic.8586

**Published:** 2024-12-20

**Authors:** Bridget Kiely, Ivana Keenan, Sonali Loomba, Natalie Mack, Vivienne Byers, Emer Galvin, Muireann O’Shea, Patrick O’Donnell, Fiona Boland, Barbara Clyne, Eamon O’Shea, Susan M. Smith, Deirdre Connolly

**Affiliations:** 1 Department of General Practice, Royal College of Surgeons, Ireland; 2 University of Medicine and Health Sciences, 123 St Stephens Green, Dublin 2, Ireland; 3 Irish College of GPs, Lincoln Place, Dublin 1, Ireland; 4 Royal College of Surgeons, Ireland; 5 Royal College of Surgeons in Ireland University of Medicine and Health Sciences, 123 St Stephens Green, Dublin 2, Ireland; 6 Environment Sustainability and Health Institute, Technological University Dublin, Dublin 7, Ireland; 7 Public Health & Primary Care, Trinity College, Dublin, Ireland; 8 Graduate Entry Medical School, University of Limerick, Limerick, Ireland; 9 Data Science Centre, Royal College of Surgeons, Ireland; 10 Department of Public Health and Epidemiology, Royal College of Surgeons, Ireland; 11 School of Business and Economics, University of Galway, Galway, Ireland; 12 Discipline of Public Health and Primary Care, Trinity College, Dublin, Ireland; 13 Discipline of Occupational Therapy, Trinity College, Dublin, Ireland

**Keywords:** social prescribing, multimorbidity, primary care, link workers, process evaluation

## Abstract

**Background:**

Social prescribing link workers support patients to connect with community resources to improve their health and well-being. These roles are prominent in policy, but there is limited evidence on what support is provided by link workers and what factors influence implementation of link worker interventions.

**Methods:**

A convergent, mixed methods process evaluation of an exploratory randomised trial of a one-month general practice-based link worker intervention targeting adults with multimorbidity in deprived areas. Qualitative data from interviews with 25 patients, 10 general practitioners, 10 link workers and eight community resource providers were thematically analysed and integrated with quantitative data to explore implementation, adaptations, context and mediators.

**Results:**

GPs reported recruitment challenges related to complicated research documentation and COVID-19 related workload and restrictions. Despite most components of the intervention being delivered, the intervention was considered too short to support people with complex needs to connect with resources, particularly in the context of COVID-19 restrictions. Timing of the referral, location within general practice and link workers’ person-centred approach facilitated the intervention.

**Conclusions:**

For future evaluations, recruitment procedures need to be simplified and integrated into everyday practice. For patients with multimorbidity, a longer intervention is indicated to achieve connection with community resources.

## Introduction

Multimorbidity, defined as a person living with two or more chronic conditions, is increasing in prevalence and presents challenges for individuals, clinicians and the healthcare system [1]. In areas of social deprivation, multimorbidity develops at a younger age and presents with more complex mixes of physical and mental health problems placing strain on primary care services [2]. Randomised trials evaluating interventions aimed at improving health outcomes for people with multimorbidity have had mixed results to date [3]. Not surprisingly, complex interventions face challenges in effectiveness evaluations due to the need for flexibility in how interventions are implemented and the influence of the specific context and limitations of outcome measures used to capture the range of potential impacts [4,5]. The UK Medical Research Council [MRC] recommends conducting process evaluations to improve the evaluation of complex interventions [6,7].

Social prescribing link workers are non-health or social care professionals who support people to connect with community resources to improve their health and well-being [8] and have the potential to address some of the individual and system challenges associated with multimorbidity. Social prescribing is a broad term used to describe any process that connects individuals with non-clinical supports and activities in their community. There are three stages to the process; access to the social prescribing service; engagement [usually involving link worker support] and activities [the resources or activities participants connect with in their community] [9]. Link workers are a key component to successful engagement and adherence to prescribed activities [10]. The link worker role is becoming increasingly common as part of wider policy initiatives to support person centred care and reduce pressures on general practice [11].

Social prescribing link worker interventions are complex; they target a range of behaviours, act on a number of levels [individual, practice, and community], and have a high degree of flexibility in how they are implemented based on individual needs and local context [7]. This heterogeneity makes establishing an evidence base on their effectiveness challenging and currently there is insufficient evidence to determine effectiveness or the usefulness of different models [12]. Therefore, when evaluating link worker interventions, it is important to identify what is implemented, for whom, and in which specific context [13,14]. Furthermore, the COVID-19 pandemic affected how social prescribing is delivered, with more remote support and reduced availability of in person community resources [15,16]. Evidence is still emerging on how link workers adapted their ways of working during the pandemic and it is important to understand the potential positive and negative impacts, given that some changes to working practices may persist.

To address this evidence gap we conducted an exploratory randomised controlled trial [RCT] and cost utility analysis of general practice-based social prescribing link workers for people with multimorbidity in areas of urban deprivation between June 2020 and January 2021, during the COVID-19 pandemic, the results of which have been published separately [17,18]. Given the complex nature of the intervention, we conducted a mixed methods process evaluation with the overall aim of investigating the implementation process, including the influence of the specific context and COVID-19 pandemic on this, and potential mechanisms of impact of the LinkMM intervention.

The specific objectives of this process evaluation paper are to:

1. Describe how the intervention was implemented for patients.
2. Describe any adaptations to the intervention, including those related to COVID-19.
3. Explore the influence of context on, and barriers and facilitators to, implementation.

## Methods

### Process evaluation design

A convergent, mixed methods [involving the collection of different but complimentary data] process evaluation, with integration using triangulation at the results stage was undertaken [19]. The methods were informed by the MRC guidance on conducting process evaluations of complex interventions [6]. Quantitative data on implementation fidelity [whether the intervention was delivered as intended], dose [how much “intervention” patients received], and reach [demographic characteristics of those who received the intervention] was integrated with qualitative data on implementation, context and mediators from semi-structured interviews with patients, link workers, general practitioners [GPs], and community resource providers [CRPs] to produce a more detailed and nuanced account of the implementation process.

Ethical approval was gained from the Irish College of General Practitioners Ethics Committee. Written consent was acquired from all participants in advance of interviews and confirmed verbally at the time of interviews.

### Public Patient Involvement [PPI]

PPI was in the form of a patient advisory group that advised on this project and three others funded as part of a collaborative doctoral award [CDA] in multimorbidity. All group members had lived experience of multimorbidity. The panel advised on recruitment materials for the RCT and interview schedules for patient interviews. Further details are available in previously published papers [17,20], an evaluation of PPI involvement in the CDA [21] and a GRIPP 2 form [22] [Supplemental Material].

### Setting

The intervention and trial protocol have been described in detail elsewhere [20]. Briefly, the intervention involved social prescribing link workers located in urban general practices, who were members of the Deep End Ireland GP network [a voluntary network of GPs who self-identify as working in deprived areas] [23]. Thirteen general practices, located in urban deprived areas in Ireland participated in the trial. [See Supplementary Table 1 for practice details]. Six practices shared a link worker; the remaining practices had an individual link worker.

**Figure 1:**
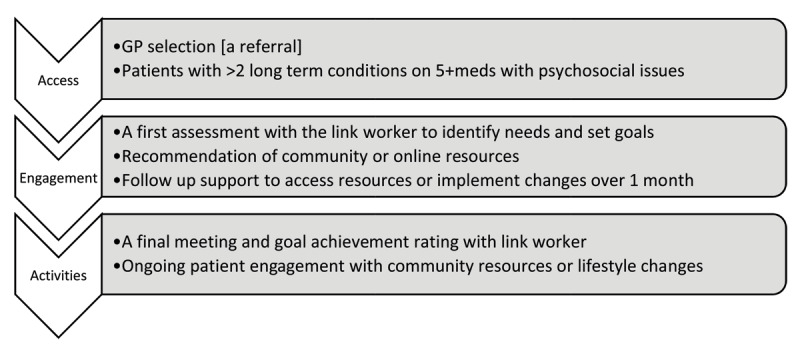
Key components of the intervention.

The process and key components of the intervention, informed by existing realist reviews of social prescribing [14,24] and input from existing social prescribing services [17], are summarised in Figure 1 and expanded in a logic model, outlining the assumptions, activities and expected outputs and outcomes of the intervention, and a TIDieR checklist [25] [Supplemental Material].

The trial employed randomisation at the individual level with a wait-list control. All participants completed baseline data collection prior to randomisation. The control group received usual care while the intervention group met the link worker. After one month all participants completed follow up data collection. The control group then met the link worker once and received a personalised list of resources. To facilitate the wait list control, link worker support was time bound. The initial intention was to provide three months of link worker support for the intervention group. However, the start of the trial was delayed by the COVID-19 pandemic and it was a requirement of the funding that the original planned number of participants were recruited, despite the shortened duration of the trial overall. In an attempt to achieve this, link worker support was reduced to one month.

### Sample

Patients recruited to the RCT were adults, aged 18 years or over, with two or more chronic conditions, attending a general practice located in a deprived urban area and deemed suitable for the intervention by their GP. For quantitative data on demographics and patient reported outcome measures [PROMs] all intervention group patients were included. For quantitative data on community resource use at one-year post intervention, all recruited patients [intervention and control] were contacted.

For qualitative data collection, intervention arm patients were sampled purposively to obtain at least two patients from each intervention site and to reflect gender and age characteristics of all trial patients. The qualitative sample size was driven by information power to capture variations in local and individual implementation [5].

All thirteen participating GPs and ten link workers were invited to interview. Each link worker was asked to provide the contact details of at least two CRPs with whom they had worked closely to provide a convenience sample of CRPs.

### Data Collection

Demographic data and patient reported outcome measures were collected from trial baseline questionnaires. Quantitative data on implementation and community resource referrals were collected from a Microsoft Access [26] client management database [CMD], used by link workers to keep records of their interactions with patients. Link workers recorded the main type of support provided at each follow up contact, based on categories of ‘synthetic social support’ which is categorised as: informational [providing information about services or resources], emotional [listening and providing emotional support], appraisal [coaching and problem solving] and instrumental [doing things for patients or accompanying them to activities] [27]. Data on community resource use were collected via a questionnaire mailed to all patients one year after intervention completion. This included intervention and control group patients, who had met with the link worker and been recommended local resources by that time.

Qualitative data were collected using semi- structured interviews conducted by telephone. Topic guides focused on factors related to implementation informed by the MRC guidance on process evaluations and based on the logic model of expected activities and outputs [6,28].[See Supplementary Material for topic guides]. Interviews were conducted by BK, who is a female GP with previous experience in qualitative interviews. BK also received additional training from BC, a female senior researcher with significant qualitative research experience. All interviews were audio recorded. No field notes were made. Patient interviews were conducted six to eight weeks after they completed the intervention. GP and CRP interviews were conducted within three months of trial completion [January 2021] between February and April 2021. Link worker interviews were conducted in January 2021.

Data sources used to address various areas of implementation, context and mediators affecting implementation are summarised in Supplementary Table 2.

### Data Analysis

Quantitative data on practice and participant demographics, and implementation data from the client management database were analysed using descriptive statistics in Stata version 15 [29]. Data on number and type of community resources were analysed using Microsoft Excel.

Semi-structured interviews were audio recorded, transcribed verbatim by a third-party transcription company, checked and deidentified by the research team, and uploaded to NVIVO to facilitate a thematic analysis [30]. The patient, link worker, GP and CRP data were analysed separately due to differences in topics covered in interviews. BK and IK [an independent female researcher with a background in sociology and primary health care research] listened to the audio files, read and re-read transcripts to familiarise themselves with the data. BK, IK, and DC [a senior researcher with a background in occupational therapy and qualitative expertise] coded a sample of interviews independently, applying a hybrid inductive and deductive approach [31] using the MRC framework areas [implementation, context, mechanisms of action and mediators] as a guide. The hybrid approach complemented the research questions, by allowing the MRC framework to provide an overall structure through the deductive analysis, but also allowed for additional themes and subthemes to be identified using an inductive approach [31,32]. Initial codes were agreed in discussion with DC. BK and IK then coded an equal division of the remaining interviews independently. BK, IK and DC met frequently during this process to review codes. Rigour was ensured by checking independently coded transcripts and reflective discussion enhanced by the researchers’ different perspectives. Codes were labelled and organised into major subcategories and categories, which were then organised under themes outlined by the MRC framework. The final categories and themes were reviewed, discussed and agreed upon by all members of the process evaluation study team.

### Integration

Qualitative and quantitative data were analysed separately as described above and integrated by BK using a triangulation protocol [19], where the different data sources were reviewed and assessed for agreement, partial agreement, dissonance, or silence under the main themes and categories.

## Results

### Participants

There were 238 participants with data available for analysis in the RCT. Of the 123 randomised to the intervention arm, 102 met the link worker at least once. There was a low response rate [28%] to the one-year survey on community resource use [66/238], with demographics of respondents being similar to the intervention group. Of the 42 patients contacted for interview, 25 accepted, eight declined and nine were unavailable in the study period. The main reasons for declining were lack of time, health issues and language barriers.

Eleven of the thirteen participating GPs were interviewed. One had moved to a new role by the time of interviews and another was unavailable due to work commitments. All ten link workers were interviewed. Eight CRPs were interviewed. Two link workers did not provide contacts for any CRPs and the remainder only one contact as they did not develop a close working relationship with many providers due to COVID-19 restrictions. See Table 1 for interview participants profile and interview duration.

**Table 1:** Interview participants profile and interview duration.


PARTICIPANT GROUP	n	CHARACTERISTICS	MEAN INTERVIEW DURATION, [RANGE] MINUTES

**Quantitative data sample**			

Intervention group patients	123	65% female 43% aged >65 87% GMS card holders 85% met LW at least once, median 3	N/A

One year follow up patient survey	66	67% female 41% aged >65 81% GMS card holders 58% intervention group 83% met LW at least one, median 2	N/A

**Qualitative data sample**			

Patients [Quote ID: GPxxPxx]	25	69% female 31% aged >65 88% GMS card holders	21 [11,12,13,14,15,16,17,18,19,20,21,22,23,24,25,26,27,28,29,30,31,32,33,34,35,36,37,38,39]

Link Workers [Quote ID: LWxx]	10	80% female Professional background Health promotion [1] Psychology [3] Social care [3] Addiction counselling [3]	68 [55–86]

General Practitioners [Quote ID: GPxx]	11	45% female All had >10years experience	36 [28,29,30,31,32,33,34,35,36,37,38,39,40,41,42,43,44,45,46,47,48]

Community Resource Providers [Quote ID: CRPxx]	8	100% female Organisation type HSE Self-management programme [1] Employment agency [1] Community partnership [5] Women’s group [1]	26 [17,18,19,20,21,22,23,24,25,26,27,28,29,30,31,32,33,34,35,36,37,38]


GMS = General Medical Services. The GMS scheme is a means-tested system that provides public medical care to approximately 40% of the Irish population. It provides eligible patients with free GP visits, free hospital care and free medications. HSE = Health Service Executive.

### Implementation

#### Recruitment

Trial recruitment was slow and the overall recruitment rate [19%] was lower than anticipated based on previous trials [50%] [33] and the pilot study [73%] [17]. The qualitative data showed that complicated research documentation, the COVID-19 pandemic and unfamiliarity with the linkworker role contributed to lower-than-expected recruitment rates.

*“I did help one man fill out the form, and it took us 40 minutes.” GP02b*

*“at least five or six people who said I wouldn’t mind doing it, but I just can’t face anything or didn’t want to come out because of COVID.” GP04*

Trust in the GP facilitated recruitment and helped overcome any hesitation related to unfamiliarity with the role.

*“I think because they have such faith in their GPs and their doctor you know that they think okay this is something that might benefit me even though they might not have been sure what it actually entailed.” LW05*

For additional supportive quotes please see Supplementary Table 3.

#### Reach

##### Demographic profile of patients

Patients recruited to the main trial experienced multimorbidity and deprivation as indicated by their mean medication counts [12] and GMS eligibility [87%]. Patients who did not meet the link worker were more likely to be on ten or more medications [81% vs 55%, p = 0.02] and a higher proportion of patients who did not meet the link worker had anxiety scores in the severe range [HADS Anxiety >15] [48% vs 21% p = 0.009]. Otherwise there were no differences between those who met the link worker at least once and those who did not. [Supplemental Table 3]. The qualitative data was in partial agreement, but indicated a more nuanced picture, with GPs judgement playing a significant role in who they chose to invite and different opinions from GPs, linkworkers and CRPs about whether suitable patients accessed the intervention.

##### Different opinions on suitability of patients

There was significant variation in how many patients the GPs identified as being suitable for the intervention ranging from 10% to 69% of all patients on 5+ medications [Supplementary Table 1]. Some of this variation could be related to patients identified in the initial search not meeting inclusion criteria. It may also be explained by GPs judgement about whether the person might engage influencing their decision to invite patients to participate. GPs cited mental health problems, complex health issues, frequent attendance and social isolation as reasons they invited patients. They also identified patients that they found generally challenging and complex.

*“Patients that we found challenging and difficult and felt that we weren’t doing what we could be doing for them or that it was to help us with them.” GP03*

GPs felt that those who might benefit most were hardest to engage and did not sign up:

*“I did phone a lot of people to try and get them to link in with the link worker, but the people that I most wanted to do it, didn’t do it.” GP02a*

While the GPs were often selecting their more challenging patients, from the link workers perspective some of the cases were unsuitable due to complexity, related to social circumstances or mental health issues. The link workers felt that some complex patients were not appropriate for a link worker given the inadequacy of their own training to deal with such complexity and the short intervention duration.

*“I can give you an example, that we have people that have very deep mental issues, and some of the link workers want to really help, but we don’t have that preparation. And [GPs] don’t realise that, and they are confused [with] the limits we have as a link worker.” LW04*

In other cases, the GPs were impressed with the link workers ability to manage complex patients and support them with a different approach.

*“I was really impressed with her management of some quite difficult patients. And coming at it from a different angle to the medical model that we would be trained in.” GP04*

CRPs felt link workers based in the GP practices allowed isolated people to engage with their services. While this required additional support from the link worker or CRPs themselves, they felt the patients referred to them were suitable and benefitted.

*“they wouldn’t be participants that would ring up and book themselves in, the anxiety, the social inclusion, the very shyness from them.” CRP01*

#### Link worker and patient meetings

##### Link worker and patient meetings: fidelity and dose

Over 80% of patients completed the key components of the intervention. Mental health was the most common area patients set goals in at 25% [20/81] of all primary goals, followed by diet and physical activity at 18% [15/81], social and community connections [15% [12/81]], and self-management of physical health conditions [10% [8/81]]. Overall 51% of participants rated their primary goal as achieved. (Table 2) Goal achievement ratings were highest for diet and physical activity [67% achieved], social and community connections [50% achieved] and lowest for mental health [40% achieved] and self-management of physical health conditions [38% achieved]. [Supplementary Table 5]. There was no difference in primary outcomes between those who achieved their goals and those who didn’t, using a mixed effects regression model controlling for baseline data and with GP practice as a random effect. Those who achieved their goals were more likely to have an increase in their self-rated health as measured by the EQ-5D-5L VAS. However, this is an exploratory analysis only, based on small numbers [n = 68]. Goal attainment scaling has been found to be correlated with a range of functional measures for rehabilitation patients [34,35].

**Table 2:** Fidelity of implementation.


FIDELITY	% [n/N]

First assessment completed	85% [102/120]

Goals set	80% [82/102]]

Community resources recommended	86% [88/102]

Final assessment completed	83% [100/120]

Primary goal achieved, Yes	51% [41/81]

Number of Goals Set [Median[range]]	2 [1–20]

Number of community resources recommended [Median [range]]	3 [1,2,3,4,5,6,7,8,9,10,11,12,13,14,15,16,17,18]

Number of follow up meetings [Median [range]]	3 [1,2,3,4,5,6,7,8,9,10,11,12,13,14,15,16,17,18,19,20,21]


On average link workers spent three hours communicating directly with patients, including the first assessment and follow ups. They estimated researching resources took an additional eight hours per patient. [See Table 2 and TIDiER checklist for further details]. Individual link workers had different approaches to follow ups, with some doing weekly calls and others more frequent communications tailored to the needs of the participant. Patients were generally happy with frequency and methods of communication from the link worker and felt that the follow-ups were important for relationship building and trust.

*“he was always in contact sending me WhatsApp messages and stuff like that and asking how I was and was I keeping to a routine.” GP08P19*

Link workers recorded the main type of support they provided during each contact in the CMD. The majority of support was informational [62%] where the link worker provided details on a local community resource, a link to a website or healthy lifestyle advice. Patients valued tailored support, but at times were overwhelmed with information.

*“Like how many pages? One, two, three, four, five… I have six pages. MABS as well. Six pages of information here that I haven’t quite read yet, but [laughing] yeah so…” GP04P36*

While the information was important the appraisal type support [21%] provided by link workers was also key in patients engaging with change.

*“The encouragement and the information but the encouragement did was a huge help and I wouldn’t have done it if I hadn’t of… I wouldn’t have done it on my own.” GP09P47*

Patients also benefitted from instrumental support [3%] particularly with digital connections.

*“she actually arranged to go on Zoom with me at home. And it was great” GP05P36*

Link workers reported they provided significant emotional support [21%], alongside other forms.

*“emotional support is huge, yeah so although you still might be evaluating something you still might be giving somebody information on something, very often that’s tied in with offers of emotional support as well.” LW08*

For additional details and supportive quotes please see Supplementary Table 3.

##### Adaptations: Remote link worker support

While link workers made every effort to meet patients face-to-face at least once, there were times when they had to deliver all or the majority of the intervention over the phone due to COVID-19 restrictions. Link workers reported how this made building relationships more difficult and the whole process took longer than face-to-face delivery. They felt face-to-face delivery was more effective to build rapport, obtain information and motivate people.

*“I felt when I did the face-to-face in the [other practice] it was like completely different. I felt there was much more, it was just much easier to get information, to build rapport.” LW03*

Link worker integration was negatively impacted by remote working in those practices where it was necessary to comply with COVID-19 restrictions.

*“I had very little contact with my GP practice, so I suppose because of COVID-19, it was very difficult, and I understand it.” LW10*

##### Barrier and facilitators to intervention implementation

###### Facilitators

Facilitators to the intervention included location within the general practice, the timing of the intervention and personal attributes of the link worker.

Location in the GP practice was convenient for patients and helped to legitimise the intervention and link worker role, both for patients and GPs.

*“It was great that it was in [my doctor’s] room because I trust [my doctor] because he’s my doctor for the last 20 years. So, that was kind of comfortable as well meeting LW in that room.” GP04P25*

*“I think it is essential that you know the link worker. If they are dealing that close with your patients, you need to know them*.” *GP06*

Having a practice-based link workers improved communication between GPs and link workers particularly around complex cases.

*“if I met somebody and it was a kind of complex case, for example I might maybe text [GP] and say look, have you got five minutes at some stage” LW04*

There were also benefits to the practice such as fostering a greater awareness of community resources and fostering a more holistic approach.

*“it brought up a different idea. So, it brought up the concept of oh, services in the community that we’re not availing of.” GP05*

Patients referred at a time when patients had an active issue that the link worker could support them with reported great benefit. For others the referral wasn’t well timed, because they had competing priorities or felt they would have benefitted from support at the time of a new diagnosis.

*“my son’s depression and living alone and all those things was a concern at that time, so it was just the right time that I met up with her really.” GP01P05*

Finally, personal attributes of the link worker meant patients felt they had a non-judgemental source of support, focused on what mattered to them and providing person centred care.

*“someone listening to me rather than like everybody telling me what to do” GP04P25*

###### Barriers

The limited time frame was a major barrier to the intervention. It took time for link workers to establish a relationship that would allow for a meaningful process of goal setting and providing support. Often patients had not connected with resources when link worker support ended, which could make ending the intervention difficult and potentially harmful for more vulnerable patients.

*“Yes, longer. Yeah. Yeah. I don’t know how much more work we could have done together, or how far we could have gotten, but yeah, I was… I’m devastated at losing it” GP04P36*

See Supplementary Table 3 for more details and supportive quotes.

#### Connection with community resources

**Figure 2:**
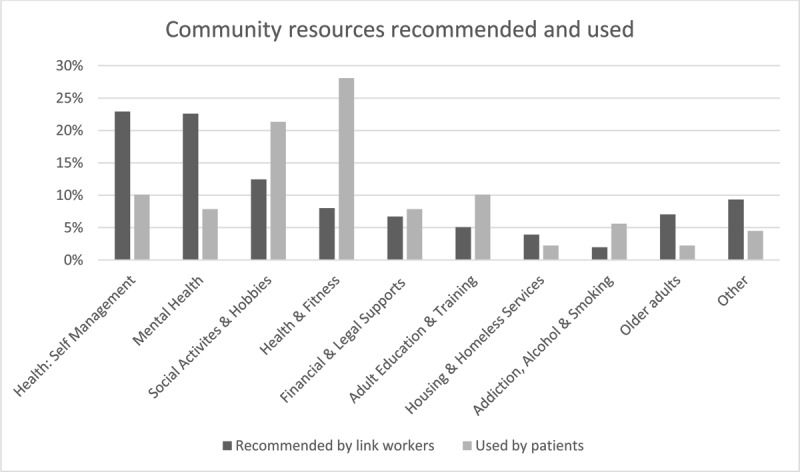
Community resource categories recommended by link workers as % of all resources [data from CMD] and used by patients [from the one year follow up survey].

Link workers mainly recommended online health self-management supports and mental health resources, but patients reported accessing supports related to health and fitness activities and social and personal hobbies as a result of link worker support in the one-year follow-up survey (Figure 2). While 65% [43/66] of respondents recalled resources recommended by the link worker, only 35% [23/66] reported contacting them and 26% [17/66] respondents said they were still engaged with resources at one year. Joining a group or club was reported by 9% [6/66] of respondents. There was partial agreement with the qualitative data. A similar proportion of patients interviewed [35% [9/26]] reported connecting successfully with resources, but they were more aligned with the link worker recommendations in that 33% [3/9] reported connecting with health condition specific supports.

##### Adaptations: Online versus in person resources

COVID-19 restrictions meant a switch to more online resources, instead of local face-to-face community resources as originally intended. Link workers supported patients interested in connecting digitally, which at times required a lot of hands on support, even helping people to change their mobile phone contracts. GPs were surprised at the people who were able to connect digitally and it changed their attitude towards their patients’ capacity.

*“Because I realise now that a lot of them can manage computer stuff that they said they couldn’t. So, I’m much more open to pushing them towards doing something online…” GP03*.

These patients experienced great benefit from support with digital access, but for others there were too many barriers.

*“this lady was all geared up to do the course and then IT, or you know, because a smartphone costs money and Internet costs money and things like that, as well as the fact that it’s alien to a lot people to use these kinds of things.” LW05*

##### Barriers to connecting with community resources

Further barriers were related to the impact of COVID-19 restrictions on available services, patients not having the time or motivation to connect, or a general lack of available resources to address some patients’ needs, such as housing and mental health.

*“there wasn’t a lot myself or [LW] could do with the country shut down” GP02aP13*

*“It is there in my mind, but I don’t really have the push to do it.” GP03P117*

*“[mental health services] are hugely under pressure now and there’s a waiting list so I would say yes that’s where there’s a huge gap.” LW08*

No community resource providers reported capacity issues and were happy to get additional referrals, however no mental health or housing service providers were interviewed.

## Discussion

This mixed method process evaluation of a social prescribing link worker intervention, identified barriers to recruitment and onward connection with community resources. Despite most components of the intervention being delivered, the intervention was too short to support people with complex needs to connect with resources, particularly in the context of the COVID-19 pandemic. Timing of the referral, link worker location within a GP practice and the person-centred approach of the link worker were facilitators to engagement with, and delivery of, the intervention.

Recruiting research participants in disadvantaged communities is recognised as challenging due to mistrust, low literacy levels and lack of understanding of research processes [36]. Studies with similar target populations reported that considerable time and effort were required to achieve recruitment targets [37].

Despite recruitment challenges the demographic profile of the patients involved indicated that people living with complex multimorbidity and social deprivation participated in the trial. However, patients who were on ten or more medications were less likely to meet the link worker, possibly related to treatment burden, which can limit capacity for lifestyle changes and making appointments [39]. Patients with mental health problems are a recommended target population for social prescribing [11,40], but the very issue patients are referred with could be a barrier to engagement as seen in our study where patients with higher anxiety scores were less likely to meet the link worker. Without the GPs invitation, many patients would not have considered themselves as potential candidates for social prescribing. Previous studies have identified the importance of the healthcare professional referrals as they legitimise the intervention [38]. However, GPs felt that their patients who needed it most, declined the GPs invitation to participate. Other studies have reported on how those who are most disadvantaged struggle to benefit from current social prescribing models [41,42].

Patients’ perception that social prescribing could help them with their current problems were identified as facilitators for engagement in this and other studies [14]. The research design in our study meant that GPs phoned potential participants, rather than referring them when they presented with a problem that may be amenable to social prescribing. This so called “cold calling” may have led to sup-optimal timing of the referral and less engagement as found in other studies [41].

The link workers succeeded in delivering the core elements of the programme, with adaptations due to comply with COVID-19 related restrictions. They provided person centred care and achieved a comparable number of contacts to other studies [33,43]. Emotional and appraisal type support were important components of person-centred care as patients felt heard and supported. Patients valued the information provided by the link workers, however many did not contact resources. Contacting resources on behalf of patients was one way link workers overcame this barrier. However, the intervention was challenging to deliver within a one-month time frame, especially for a population who had complex needs. This, along with reduced availability of in-person resources due to COVID-19 related restrictions, meant there was lower onward connection with community resources compared to longer interventions [33].

There was a discrepancy between supports recommended by the link workers and those patients reported accessing at one year. This is possibly due to a lack of availability of services in-person, or that resources like chronic disease self-management courses frequently recommended by link workers as they were one of the few resources available on-line durng the pandemic, only ran for a limited period. Patients reported more success in achieving lifestyle related goals. This could be because these were more achievable within the time frame of the intervention and context of COVID-19 restrictions and more sustainable in the longer term. It may also indicate a “lifestyle drift” seen in shorter interventions, where the focus tends to be on individual health related behaviours rather than addressing underlying psychosocial issues [44]. Few patients reported ongoing engagement with resources or having joined a group at one year. This suggests that ongoing social connections, a key process of social prescribing when considered through the “social cure” theory, had not developed [45].

Finally, while the impact of the COVID-19 pandemic may be seen as an unmodifiable and isolated event, there are lessons to be learned about the limitations of remote telephone interventions compared to face-to-face interactions. Recent studies conducted during the COVID-19 pandemic have also reported the challenges of remote and digital connections [15,16]. That said support with digital access was transformative for some patients in this study.

### Strengths and limitations

Our process evaluation drew on multiple quantitative and qualitative data sources and perspectives to give a comprehensive picture of the implementation process. We provided detail on the number of contacts with the link worker and community resources recommended to, and engaged with, by patients, data frequently lacking from other evaluations [13,46]. Integrating quantitative data on implementation with qualitative data from interviews allowed more in-depth exploration of findings than would have been possible with either method alone. Often evaluations only look at individual perspectives, but by considering perspectives from different roles, we were able to confirm findings, such as the importance of the GP role in referral and engagement.

However, we have limited insight into those who did not take part and are relying on qualitative data from the GPs perspective to explore recruitment challenges. There were no observed interactions between GPs, link workers and patients to assess fidelity and so we are relying on the quality of documentation of activity by the link workers and interviews to assess this. The length of the intervention limited the potential to explore mechanisms of action in detail. Interviews were conducted shortly after intervention completion and so there was no opportunity to further explore findings from the one-year follow-up survey such as the differences between recommended and used community resources.

### Implications for practice, policy and research

Our findings suggest that a one-month intervention is unlikely to be suitable for people with more complex needs. If link workers are to support more complex patients, the impact on their work-load will need to be considered so that more time can be allocated to these more difficult cases in particular. Similar interventions that reported positive outcomes were between six and 24 months duration [47,48].

While link worker location varies depending on local context, some time in general practice is recommended to facilitate improved understanding of the link worker role and communication between the GP and link worker around suitable referrals and availability of community resources. Our study showed the vital role that the GP played in facilitating individuals who may not have otherwise accessed social prescribing to participate. It also showed how the link worker intervention had potential to deliver person centred care if patients were referred at the right time [when they had an active psychosocial issue], to the right person [a link worker], in the right place [general practice]. This is in line with a drive towards integrated and community care in Ireland’s main health strategy “Slaintecare: Right care. Right place. Right time” [49]. Link workers have been introduced at a community level in deprived communities as part of a healthy communities initiative, which is part of the implementation of this strategy and aims to reduce health inequalities related to life style factors [50].

However, there is a risk that social prescribing interventions could reinforce health inequalities if the most vulnerable individuals are excluded or if support is not of significant intensity or duration to address the complexities of their needs. Link workers are not a replacement for comprehensive mental health services or upstream investment in the social determinants of health [44,51]. Policy makers should be clear on their expectations of what social prescribing can and cannot achieve in order to avoid shifting focus on addressing health inequalities from system and structural levels to an individual level [42].

The evidence base for social prescribing is expanding, but gaps around effectiveness remain [12]. The issue of timing of referral and duration of support in this study was in part due to our wait-list control design and individual randomisation. A pragmatic cluster randomised trial design or stepped wedged design would allow for flexibility of duration of link worker support and for GP practices to identify suitable patients during routine practice, including chronic disease reviews [52]. Future evaluations also need to consider a longer intervention and additional time and supports for recruitment to ensure those who could benefit most are not excluded. The MIDAS trial is an on-going cluster randomised trial investigating the effectiveness of the addition of six months of social prescribing link worker support to routine chronic disease reviews in general practice and applies lessons learned from this study. It also includes a parallel cost utility analysis and process evaluation [53]. Other approaches to evaluation include realist evaluations and social return on investment analysis, which can provide rich context specific data on mechanisms of action and potential social value [54].

## Conclusion

Link worker support was successfully implemented with adaptations due to COVID-19 restrictions, but this did not always translate into connection with community resources. This was likely because the one-month intervention was insufficient to engage and support people with complex needs, which was exacerbated by the impact of the COVID-19 pandemic. Future evaluations should consider a longer intervention and additional supports for recruitment to ensure adequate inclusion of, and support for individuals with complex needs.

## Additional File

The additional file for this article can be found as follows:

10.5334/ijic.8586.s1 Supplementary Tables. Tables 1 to 5.
