# Supplementary material for: Implementing a General Practice-Based Link Worker Intervention for People with Multimorbidity During the Covid-19 Pandemic- a Mixed Methods Process Evaluation of the LinkMM RCT
Source: Int J Integr Care. 2024 Dec 20;24(4):16. doi: 10.5334/ijic.8586 (PMC11661012; doi:10.5334/ijic.8586)
Supplement: Supplementary Tables. — Tables 1 to 5. [file ijic-24-4-8586-s1.pdf]

Supplementary Table 1. GP demographics, selection and recruitment rates

ID	Patients* living in deprived area	GMS list size	Patients on 5+meds	Patients deemed suitable for LW	Patients who were sent packs (from GP records)†	Recruited	Recruitment rate
		n	n (%)	n (%)	n	n	%
**GP01**	34%	3865	784 (20%)	538 (69%)	85	21	25%
**GP02A**	68%	2136	140(7%)	90 (64%)	70	17	24%
**GP02B**	73%	2000	618 (31%)	61 (10%)	9	7	78%
**GP03**	39%	2676	1459 (55%)	270(19%)	105	32	30%
**GP04**	77%	4468	568 (13%)	110 (19%)	110	12	11%
**GP05**	45%	2720	178 (7%)	100 (56%)	67	22	33%
**GP06**	37%	1264	*329 (26%)*[1]	*102(31%)*[1]	Missing data	34	Missing data
**GP07 (A&B)**	Missing data	2285	1127 (49%)	350 (31%)	50	14	28%
**GP08**	24%	3763	1030 (27%)	142 (14%)	30	14	47%
**GP09**	53%	3400	744(22%)	198 (27%)	136	35	26%
**GP10**	88%	4510	774 (17%)	112 (14%)	85	19	22%
**GP11**	28%	1544	533 (35%)	70 (13%)	27	13	48%
**TOTAL ELIGIBLE PATIENTS**[1]				2103			

*All registered patients. GMS: general medical services. † due to recruitment challenges there was variation in approaches both between practices and over time, with some practices phoning patients in advance and only sending recruitment packs to patients who said they would be interested. Documentation of this was incomplete. [1] Data were not available from one GP practice. To estimate the total number of eligible patients the mean proportion of patients on 5+ meds and deemed suitable for the intervention were calculated from the other practices

Supplementary Table 2. Overview of questions and corresponding data sources

Question?	Data Source
**Implementation: reach**	
Whom did GPs select to take part? What barriers to recruitment did they encounter?	Qualitative: GP Interviews
Why did patients decide to take part?	Qualitative: Patient Interviews
Did patients recruited to the trial represent the target population?	Quantitative: Demographics and baseline scores of trial patients
	Qualitative: Link worker, GP and CRP interviews
**Implementation: fidelity, dose and adaptations**	
Did patients meet with link workers and complete key components of intervention?	Quantitative: Proportion of intervention patients who had an initial and final meeting, were referred to a community resources, set goals and achieved goals from CMD
How much support did link workers provide?	Quantitative: Number of follow up contacts and communication method from CMD
How did link workers provide support?	Quantitative: Proportion of encounters where informational, appraisal, emotional or instrumental support was provided
	Qualitative: Patient and link worker interviews
What adaptations were made?	Qualitative: Link worker interviews
What types of resources were patients referred to?	Quantitative: Categories of resources recorded in CMD
Did patients engage with resources?	Quantitative: Proportion of patients still engaged with resources and categories of resources engaged with from one-year follow up survey.
	Qualitative: Patient interviews
Were community resources available, in person or online?	Qualitative: Patient, link worker and CRP interviews
**Context**	
What impact did COVID-19 have on implementation?	Qualitative: Patient, link worker, GP and CRP interviews
What impact did the location of the link worker have on implementation?	Qualitative: Patient, link worker, GP and CRP interviews
**Mediators**	
What were the barriers and facilitators to the intervention?	Qualitative: Patient and link worker interviews

GP= General Practitioner. CRP= Community Resource Provider. CMD= Client Management Database

Supplementary Table 3. Additional supportive quotes

**Theme: Recruitment and engagement**	
**Summary of qualitative findings**	**Supportive quotes**
<u>Trust in the GP</u><br>Patients (n=14) described getting involved because it had come from a trusted source- their GP. Patients (n=9) reported they would not have seen themselves as candidates for social prescribing otherwise.	*" the GP phoned me personally and asked me would I fill it in, then yes, I felt confident and that, so that is the reason I filled it in." GP01P05.*<br><br>*"I wouldn't think that would be for me, I would think that that's for people that are really sick or something." GP11P09*
The link workers (n=5) also reported that trust in the GP was evident in their interactions with patients.	*"I think because they have such faith in their GPs and their doctor you know that they think okay this is something that might benefit me even though they might not have been sure what it actually entailed." LW05*
<u>Patient unfamiliarity with social prescribing</u><br>Both GPs and patients reported a lack of familiarity with the link worker role among patients. Some GPs (n=2) found this was a potential barrier to recruitment.	One GP reported a patient saying *"I don't really want someone getting involved in my affairs"* and the GP felt it *"probably goes back to the old thing of social workers calling around." GP05.*
Link workers (n=8) reported that many patients needed to be reassured about the role, indicating some people got involved despite not having a clear understanding of the role.	"*just explain to them a little bit about what the role is and kind of try and ease them into it because it can be a little bit daunting and they mightn't… There were a few people that would have agreed to taking part in it but they weren't really sure what they were taking part or what it was really about." LW04*
<u>Recruitment challenges related to research documentation</u><br>GPs (n=8) reported that the paperwork was off-putting for many of their patients, who they perceived had lower literacy levels than the general population.<br><br>GPs also reported this based on their experience assisting some people with the study consent and baseline questionnaire documents, which they believed were complicated and time consuming. There are no data from interviewed patients or link workers on this topic.	"*the problem was that they would all say that they would do it, you see. They wouldn't say that they couldn't face the form." GP03*<br><br>*"I did help one man fill out the form, and it took us 40 minutes." GP02b*
<u>Impact of COVID-19 restrictions on recruitment</u><br>Patients were reluctant to get involved during COVID restrictions, as reported by GPs (n=8); patients didn't want to come to the GP or couldn't envision how the intervention would work with restrictions.	*"at least five or six people who said I wouldn't mind doing it, but I just can't face anything or didn't want to come out because of COVID." GP04*
The GP who had taken part in the pilot noted the difference. (n=1)	*"the recruitment process was utterly different between the pilot and the project. They were very*

	wary about coming down to the practice" GP03
GPs were very busy and given recruitment was time-consuming, this was exacerbated by COVID (n=4).	*"obviously COVID was the biggest one because we just had to make so many other changes in the practice and there was so much logistically going on that finding any time to do anything outside of that was really very challenging." GP07b*
There was dissonance in patient interviews, as those patients who decided to get involved were not put off by the COVID-19 restrictions.	*"No, it was actually great. It was something to do" GP06P55.*

**Theme: Support provided by link workers<br>Summary of qualitative findings**	**Supporting quotes**
**Informational (62%)**	
Patients valued the tailored information that they felt they would not have found on their own. (n=15)	*"She got me in touch with this fellow. I never knew he existed; this housing - so called - liaison officer. I never knew he was there. We don't know about these things."* GP06P08
At times they reported being overwhelmed by the amount of information. (n=3)	*"Like how many pages? One, two, three, four, five… I have six pages. MABS as well. Six pages of information here that I haven't quite read yet, but [laughing] yeah so…"* GP04P36
**Emotional (21%)**	
Emotional support from a non-judgemental independent source was important to patients. (n=19)	*"it was someone to go in to talk to. It was someone to confide in. It was someone who was there to assist whatever concerns that you had at that time" GP01P05*
The link workers referred to providing a lot of emotional support throughout the intervention, often alongside other forms of support, suggesting that the recorded amount underestimates the importance of this. (n=9)	*"emotional support is huge, yeah so although you still might be evaluating something you still might be giving somebody information on something, very often that's tied in with offers of emotional support as well." LW08*
Linkworkers (n=4) described supporting patients with the emotional impact of the COVID-19 restrictions. Many were socially isolated, lacked routine and were dealing with anxiety. The link worker who had worked during the pilot study (pre COVID-19) commented on this.	*"the interaction with people was really different because people were a lot more fearful and distracted and full of anxiety and worry about what was happening and yeah so hugely different interaction with people"* LW08
**Appraisal (15%)**	
Patients who achieved their goals referred to the importance of the encouragement from the link worker and how meeting the link worker helped them gain confidence to engage with other activities. (n=12)	*"The encouragement and the information but the encouragement did was a huge help and I wouldn't have done it if I hadn't of… I wouldn't have done it on my own."* GP09P47
**Instrumental (3%)**	
At interview, link workers (n=5) and patients (n=7) described different forms of instrumental support:	*"if they felt a little bit nervous about contacting a resource, or joining something new, I would have*

filling out forms, contacting agencies on patients' behalf or occasionally coordinating between healthcare providers (n=3 patients).	*offered to help them with that. So, get their permission to maybe pass on their details to the person, and they receive a call instead of making the call, which I think can be a massive help." LW10*
Digital support was mentioned by patients (n=5) and link workers (n=4).	*"she actually arranged to go on Zoom with me at home. And it was great. "GP05P36*

Theme: Barriers and Facilitators to implementation

Summary of qualitative findings	Supporting quotes
<u>Location within general practice</u>	
Location within the GP practice helped to reassure patients about the link worker.	*"It was great that it was in [my doctor's] room because I trust [my doctor] because he's my doctor for the last 20 years. So, that was kind of comfortable as well meeting LW in that room." GP04P25*
Communication with GPs, either through formal meetings (in-person or online), informal "corridor chats" or email, allowed link workers to highlight unaddressed health needs of individual patients and share knowledge about community resources.	*"We learned about more resources that were in the area." GP09*<br>*"coming at it from a different angle to the medical model that we would be trained in. I think that was a really interesting insight and a useful one." GP04*<br><br>*"if I met somebody and it was a kind of complex case, for example I might maybe text [GP] and say look, have you got five minutes at some stage" LW04*
Remote working was necessary to comply with COVID-19-related social distancing restrictions in some practices (n=2) or the practice was overwhelmed with clinical work during the pandemic (n=3). In these practices there was an absence of communication between the link workers and the GPs.	*"I had very little contact with my GP practice, so I suppose because of COVID-19, it was very difficult, and I understand it." LW10*
Link workers were also only based in the GP practices for a 6-month period. While some practices quickly established a good working relationship, some struggled within the time frame.	*"You would need at least a year, at least and a year isn't a long time really." GP01*
<u>Right time</u>	
Patients reflected on the timing of the intervention. (n=7) For some patients, the invitation was well timed, in that they had active issues the link worker could support them with.<br>For others they had competing priorities that prevented their engagement or felt they would have benefitted more at an earlier stage, particularly at the time of a new diagnosis.	*"my son's depression and living alone and all those things was a concern at that time, so it was just the right time that I met up with her really." GP01P05*<br><br>*"like even if it had it been a few years ago, I was trying to like get intervention for my son and stuff 'cause he has autism"* GP07P29
<u>Personal attributes of the link worker</u>	
Link workers described taking an informal approach to initial assessment and goal setting with patients. (n=5)	This was reflected in patients describing the link worker as *"down to earth"* (GP08P29) and *"homely"* (GP03P47).
Patients felt the link worker was someone who was	*"someone listening to me rather than like everybody*

there to listen and provide emotional support in a non-judgemental way. They contrasted this to their experiences with healthcare professionals and felt the link worker role was quite different.	*telling me what to do" GP04P25*
<u>Barriers: Limited time to provide support</u><br>Linkworkers (n=10) found patients were often not ready to set goals at the first assessment and so this was an incremental process rather than happening at the initial meeting, an adaptation to planned procedure. Link workers discussed how competing life events could derail plans and made achieving goals within the one-month time frame difficult.	*"those goals would shift and suddenly it felt like they didn't have the capacity to, you know, go back and look at my goal to, you know, engage in relaxation when they had like a hugely stressful event happen in their life."" LW07*
Patients (n=11) felt the intervention was too short and often had not managed to connect with resources at the time of interview.<br>Link workers (n=10) found ending the intervention hard because of this.	*"There wasn't really enough time with [LW] to go through any processes and things like that." GP02aP13*<br><br>*" it's probably the part of the job that I didn't enjoy, because I found that even though you may have linked somebody into X, Y or Z, I still feel a lot of stuff is up in the air because of COVID" LW02*
For two of the interviewed patients, losing the link worker after a short period was upsetting and difficult, indicating a possible adverse consequence of the intervention.	*"Yes, longer. Yeah. Yeah. I don't know how much more work we could have done together, or how far we could have gotten, but yeah, I was… I'm devastated at losing it" GP04P36*

Barriers to connecting with community resources

Summary of qualitative findings	Supporting quotes
<u>Impact of COVID-19 on services</u><br>Patients (n=9) and link workers (n=9) referred to poor availability of services due to the impact of COVID-19	*"there wasn't a lot myself or [LW] could do with the country shut down" GP02aP13*<br>*"No centres were open so that was quite limiting to them because a lot of the people were so happy to have the information about the clubs, but were sad that they couldn't get stuck in" LW09*
<u>Patients personal reasons for not connecting</u><br>Patients had competing life events or family responsibilities (n=4), were not keen to participate in groups (n=4), or lacked motivation (n=8).	*"the first time I couldn't find it and the second time I had to bring my mam into The Mater to get her chest X-rayed." GP04P25*<br>*"She wanted me to get in touch with different groups and do different things. I am not a group person. " GP02aP13*<br>*"It is there in my mind, but I don't really have the push to do it." GP03P117*
<u>Limited resources for some needs</u>	

For a small number of patients (n=4), the issues they were facing were things that the link worker was not able to help them with and they reported no perceived benefit from the intervention. Most frequently, this was housing-related.	*"Well, I live in a hostel at the minute, and I am on the housing list. And she said she would see could she do anything for me, but there was nothing she could do on that one." GP02aP62*
The link workers (n=7) felt that mental health was patients' main issue, but that there was a lack of available resources and long waiting lists for mental health services, making it difficult to make progress in this area.	*"[mental health services] are hugely under pressure now and there's a waiting list so I would say yes that's where there's a huge gap." LW08*

Supplementary Table 4. Demographic Characteristics and baseline outcome measures for those who met and did not meet the link worker

Demographic characteristics	All Intervention<br><br>**N=123**<br>n (%)	Met linkworker<br><br>**N=102**<br>n (%)	Did not meet linkworker<br>**N=21**<br>n (%)	p
**Age Group**				
18-24	2 (2)	2 (2)	0	
25-44	15 (12)	12 (12)	3 (14)	
45-64	53 (43)	43 (42)	10 (48)	
65+	53 (43)	45 (44)	8 (38)	0.86
Female	78 (65)	63 (62)	15 (71)	0.40
GMS	101 (87)	82 (80)	19 (91)	0.27
Meds 10+	73 (59)	56 (55)	17 (80)	0.03
Primary education or below	35 (30)	28 (28)	7 (33)	0.59
Employed	19 (18)	16 (16)	3 (14)	0.87
Home-owner occupied	55 (50)	46 (45)	9 (43)	0.85
Living alone	32 (27)	26 (26)	6 (29)	0.77
Other language	9 (8)	8 (8)	1 (6)	0.72
Smoker	40 (34)	33 (32)	11 (52)	0.08
Alcohol 11 units+	19 (16)	31 (30)	5 (24)	0.55
**PROM at baseline**	**Mean (SD)**	**Mean (SD)**	**Mean (SD)**	**p (mean diff 95% CI)**
EQ-5D-5l index	0.473 (0.419)	0.496 (0.388)	0.361 (0 .549)	0.89 (-.34 .074)
EQ-VAS	60 (20.3)	59.6 (21.0)	61.5 (16.6)	0.36 (-8.0 11.8)
HADS	17.6 (8.9)	17.3 (8.7)	19.6 (10.0)	0.15 (-2.31 7.09)
HADS Anxiety	9.6 (5.0)	9.3 (4.7)	11.3 (5.9)	0.06 (-.50 4.57)
HADS Depression	7.8 (4.7)	7.6 (4.7)	8.3 (5.1)	0.29 (-1.68 2.95)
ICECAP-A	0.72 (0 .20)	0.726 (0.204)	0.706 (0.196)	0.65 (-.12 .08)
PAM	53.7 (13)	53.8 (13.3)	53.7 (12.4)	0.51 (-6.47 6.35)
MM Treatment Burden	20 (20)	19.6 (19.0)	21.9 (24.5)	0.32 (-.30 .489)
Frenchay Activity	41 (8.9)	41.3 (8.6)	39.7 (10.7)	0.74 (-6.53 3.33)

Comparison of proportions using Pearson's chi squared or comparison of means using t test

Supplementary Table 5. Categories of primary goals set and % achieved per category

Category	Proportion of all goals	Achieved (n)
Mental health	25% (20)	40% (8)
Social and community connection	15% (12)	50% (6)
Healthy diet	11% (9)	78% (7)
Self-management physical health conditions	10% (8)	38% (3)
Physical activity	7% (6)	50% (3)
Personal development	7% (6)	67% (4)
Housing	6% (5)	40% (2)
IT	4% (3)	0%(0)
Finances	4% (3)	100% (3)
Employment	2% (2)	100% (2)
Other	9%(7)	43%(3)
**Total**	100% (81)	51% (41)
